# Differences in response-scale usage are ubiquitous in cross-country comparisons and a potential driver of elusive relationships

**DOI:** 10.1038/s41598-024-60465-0

**Published:** 2024-05-13

**Authors:** Esther Ulitzsch, Mirka Henninger, Thorsten Meiser

**Affiliations:** 1 IPN—Leibniz Institute for Science and Mathematics Education, Educational Measurement, Olshausenstraße 62, 24118 Kiel, Germany; 2 University of Mannheim, Mannheim, Germany; 3 University of Zurich, Zurich, Switzerland

**Keywords:** Psychology, Human behaviour

**arising from**: P. Sorokowski et al.; *Scientific Reports* 10.1038/s41598-022-26663-4 (2023).

## Introduction

Research in the social sciences heavily relies on self-reports using Likert-type rating scales, measuring attitudes, beliefs, and behavior. Cross-country comparisons using these scales build on the implicit assumption that, across countries, respondents perceive and use the scales’ response options in the same way. When this assumption is violated, observed differences in mean scores do not only reflect differences in the constructs of interest but also systematic differences in response option usage—a phenomenon referred to as response styles^1^. We believe that the relationship between country-level self-reported love experiences and modernization reported by Sorokowski et al.^2^ poses an instructive cautionary tale of how the unaccounted presence of cross-country differences in response styles may lead to potentially spurious and artifactual conclusions. To support this claim, we first briefly review the phenomenon of response styles and its implications and discuss state-of-the-art psychometric approaches for accommodating response style differences. Employing these approaches, we re-analyze the data from Sorokowski et al.^2^ and show that once response styles are accounted for, conclusions on a substantial relationship between country-level love experiences and modernization are no longer supported. We conclude with recommendations for cross-country comparisons using Likert-type scales.

## Response styles and their implications

**Figure 1:**
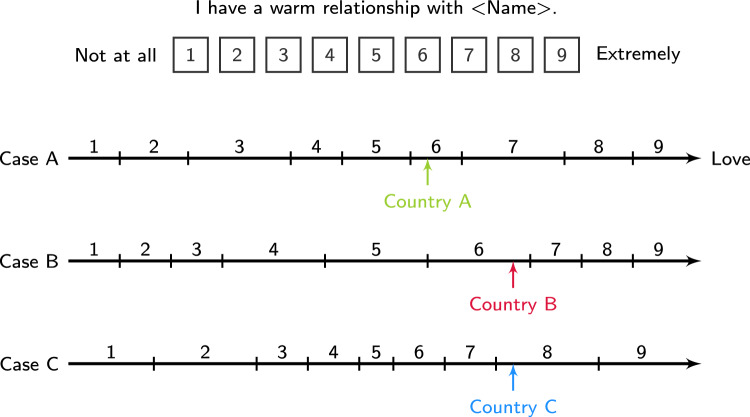
Illustration of differences in response styles on a nine-point rating scale item for three fictitious countries.

When analyzing data obtained with Likert-type scales, researchers generally assume that each response category of the scale reflects a certain interval on an underlying latent continuum of interest. The observed choice of a given response category is thus taken to indicate a respondent’s location on this latent continuum. To illustrate, Fig. 1 displays an item from the love experience questionnaire used by Sorokowski et al.^2^ with a nine-point rating scale. When respondents are presented with the item, they not only have to interpret the item content (e.g., what it means to have a warm relationship) but also the response format (e.g., what it means to choose “6” or “8”). Case A in Fig. 1 shows the average interpretation of the response format, that is, the assignment of response categories to intervals on the latent love continuum, by respondents in a fictitious Country A together with the mean location of individuals in this country.

The interpretation and use of rating scales have been shown to vary over countries^3,4^, as is illustrated by Cases B and C in Fig. 1. In Case B, respondents from Country B prefer the medium categories “4”, “5”, and “6” over broader intervals of the love continuum than respondents from Country A. Although the mean location in Country B is noticeably higher than in Country A, the observed categories corresponding with the different locations are identical (i.e., “6”) due to the different scale usage. In Case C, respondents from Country C give a more narrow interpretation of the medium categories and instead prefer the more extreme categories “1”, “2”, “8”, and “9” over broader regions of the continuum. Despite identical latent values, the mean location in Country C, therefore, corresponds with the observed response “8” rather than “6” in Country B.

If such differences in response styles are not controlled for, they jeopardize the validity of mean scores, because the same observed score may reflect different levels on the latent dimension (see Countries A and B) and different observed scores may reflect identical latent levels (see Countries B and C), potentially distorting group comparisons and correlations with extraneous variables^1,3,4^.

## A conceptual introduction to psychometric approaches for accommodating response style differences

To disentangle the measurement of latent attributes from the confounding influence of response styles, psychometric models of Item Response Theory (IRT) have been extended to accommodate response style effects in individual assessments and group comparisons. Traditional IRT models for ordinal responses like the Partial Credit Model^5^ assume that the probability of observing a given response category depends on (a) the respondent’s location on a latent trait continuum and (b) a set of item-specific threshold parameters. If the threshold parameters are increasingly ordered, each pair of adjacent thresholds defines an interval on the latent trait continuum over which one of the response categories has the modal probability, similar to the category boundaries depicted in Fig. 1. Importantly, however, traditional IRT models maintain the presumption that the threshold parameters are constant across persons and groups, leaving differences in the interpretation and use of response categories unconsidered. Extended IRT approaches, in contrast, capture such differences in terms of varying threshold parameters that mirror differences in perceived response category widths^6,7^ and allow researchers to control for response style effects in group comparisons and correlational analyses^8^.

## Love experiences and modernization revisited

Sorokowski et al.^2^ explored whether countries’ modernization levels are related to love experiences. One of the key findings was a quadratic relationship between country means of self-reported love experiences and the human development index (HDI) of 45 countries, leading the authors to speculate that “although country’s economic development generally promotes more intense love experiences, reaching a certain developmental point might reverse these beneficial love effects” (p. 6). To probe the sensitivity of this conclusion against adjustments for cross-country differences in scale perception and usage, we re-analyzed the data with (a) a multigroup PCM with country as the grouping variable, assuming that cross-country differences in chosen response categories are solely driven by cross-country differences in love experience and (b) an extended multigroup PCM accommodating cross-country extreme response style differences (i.e., a preference for outer categories). In this extended model, the extreme response style dimension is tantamount to symmetrical shifts of the threshold parameters, such that the intervals for medium response categories increase for negative values (see Case B in Fig. 1) and the intervals for extreme response categories increase for positive values (see Case C in Fig.  1) on either side of the rating scale. In both models, latent country means of love experience were related to standardized HDI values via quadratic regression. A detailed description of the employed models as well as analysis code is provided in the OSF repository accompanying this comment.

**Figure 2:**
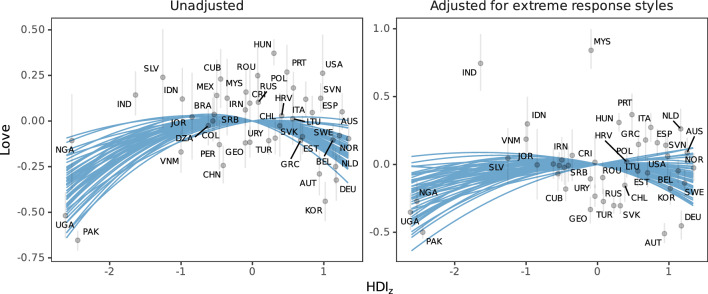
Unadjusted and adjusted love country means plotted against standardized human development index values. Gray bars give 95% credibility intervals of love country mean estimates. Blue superimposed lines give 50 posterior draws of the model-implied relationship between love country means and standardized human development index values. Note that y-axes differ in scale.

When extreme response styles were not considered, latent country means indeed exhibited a quadratic relationship with standardized HDI values ($\beta _{\text {HDI}}=-0.02$, 95% credibility interval: $[-0.10; 0.06]$, standardized coefficient: $\beta _{\text {HDI}}^{*}=-0.11$, $\beta _{\text {HDI}^{2}}=-0.06 [-0.10; -0.02]$, $\beta _{\text {HDI}^{2}}^{*}=-0.29$, see left panel in Fig. 2), mirroring results reported for scale score means in Sorokowski et al.^2^. Once extreme response style differences were taken into account, however, the quadratic relationship was weaker pronounced and no longer credibly different from zero ($\beta _{\text {HDI}}=-0.01 [-0.11; 0.08]$, $\beta _{\text {HDI}}^{*}=-0.04$, $\beta _{\text {HDI}^{2}}=-0.04$ $[-0.10; 0.01]$, $\beta _{\text {HDI}^{2}}^{*}=-0.14$, see right panel in Fig. 2). From these results, we conclude that the quadratic relationship between self-reported love experience and modernization reported in Sorokowski et al.^2^ may plausibly be driven by cross-country differences in scale usage.

## Conclusion

Countries may not only differ in typical attitudes and beliefs but also in the way respondents use the scales employed for their measurement. Based on data from Sorokowski et al.^2^, we illustrated that analyses leaving such differences unconsidered and analyses aimed to accommodate them may yield vastly different conclusions. We, therefore, recommend probing findings of cross-country comparisons for sensitivities to country differences in scale usage. In our illustration, we considered adjustments for extreme response style differences using an extended PCM. We note, however, that to the end of response style adjustments, researchers have a wide array of adjustment procedures at their disposal, each resting on different assumptions^8,9^. Since it is not evident which of these procedures yields the “best” adjustment for the data at hand^10^, we strongly advocate investigating multiple plausible implementations of response style adjustments and systematically exploring the impact of different analysis decisions on the parameter of interest^11^.

## Data Availability

Data and analysis scripts are available in the OSF repository, https://osf.io/rfy9h/.
